# Synthesis of Antimony Nanotubes via Facile Template-Free Solvothermal Reactions

**DOI:** 10.1186/s11671-016-1697-x

**Published:** 2016-11-03

**Authors:** Ruxue Li, Xiaohua Wang, Xinwei Wang, Haoran Zhang, Jingxin Pan, Jilong Tang, Dan Fang, Xiaohui Ma, Yongfeng Li, Bin Yao, Jie Fan, Zhipeng Wei

**Affiliations:** 1 State Key Laboratory of High Power Semiconductor Laser, School of Science, Changchun University of Science and Technology, 7089 Wei-Xing Road, Changchun, 130022 People’s Republic of China; 2 State Key Laboratory of High Power Semiconductor Laser, School of Materials Science and Engineering, Changchun University of Science and Technology, 7089 Wei-Xing Road, Changchun, 130022 People’s Republic of China; 3 State Key Laboratory of Superhard Materials and College of Physics, Jilin University, 2699 Qian-jin Street, Changchun, 130023 People’s Republic of China; 4 Key Laboratory of Physics and Technology for Advanced Batteries (Ministry of Education), College of Physics, 2699 Qian-jin Street, Jilin University, Changchun, 130012 People’s Republic of China

**Keywords:** Sb nanotubes, Solvothermal synthesis, Rolling mechanism

## Abstract

Uniform antimony (Sb) nanotubes were successfully synthesized via a facile solvothermal method without the need for any surfactants or templates. The Sb nanotubes are confirmed to be pure rhombohedral phase and have better crystallinity. These nanotubes show middle-hollow and open-ended structures, as well as multi-walled structures with the wall thickness of about 10 nm. Also, they have an average size of the diameter of about 50 nm and the length of about 350 nm. On the basis of the structural and morphological studies, a possible rolling mechanism is proposed to explain the formation of Sb nanotubes. It is expected that uniform Sb nanotubes can further be used in wide applications.

**Graphical Abstract:**
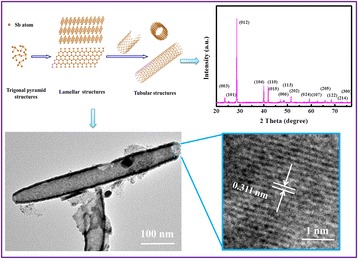
A possible rolling-formation mechanism is proposed for forming pure rhombhedral phase and high crystallinity antimony nanotubes without any surfactants or templates via a facile solvothermal method.

A possible rolling-formation mechanism is proposed for forming pure rhombhedral phase and high crystallinity antimony nanotubes without any surfactants or templates via a facile solvothermal method.

## Background

Since the discovery of carbon nanotubes, one-dimensional nanotubes have attracted much attention due to their peculiar physical properties and promising applications as interconnect and functional units in fabricating electronic, optoelectronic, thermoelectric, and electromechanical nanodevices and so on [1–4]. So far, a large number of reports have focused on the exploring whether other layered materials also can form tubular or similar nanostructures. Through constant efforts, various nanotubes have been successfully synthesized by means of their two-dimensional layer structure, such as boron nitride (BN), titanium dioxide (TiO_2_), tungsten disulfide (WS_2_), bismuth sulfide (Bi_2_S_3_), bismuth (Bi), and so on [5–9]. All the above mentioned reports indicate that substance possessing lamellar structures might be able to form nanotubes under favorable conditions. Similar to that of Bi, semimetallic antimony (Sb) has also a pseudolamellar structure and has interesting features such as low conduction band, effective mass, and high electron mobility [10–13]. In particular, the effective mass components of the electron ellipsoids in Sb are much larger than those in Bi, while the effective mass components of the hole ellipsoids of Sb are of the same order of magnitude as those in Bi [14]. Thus, the particular transport properties of the electron can be expected in one-dimensional nanostructures of Sb, which make it become an interesting system for studying quantum confinement effects [15]. Among them, Sb nanowires have exhibited the interesting electronic properties, such as surface superconductivity, extremely large magnetoresistance, and high efficiency thermoelectricity generation [16, 17]. Recently, Sb nanowires have been synthesized by the pulsed electrodeposition and vapor phase deposition in anodic alumina templates [18, 19], self-assembly on graphite templates [20], and surfactant-assisted solvothermal synthesis [21, 22]. In addition, Sb nanotubes have been synthesized via the reduction acetylacetone-assisted antimony complexes process [23]. To the best of our knowledge, the template-assisted or the surfactant-assisted methods are the most popular synthetic strategies for the synthesis of one-dimensional nanostructures of Sb, which can control the oriented growth or uniform dispersal of nanomaterials. Unfortunately, the removal of the templates will give rise to poorly defined nanostructures from complex procedure, or the existence of organic surfactant will affect the properties of the Sb nanomaterials, which consequently have limited their large-scale production and further potential applications. However, there have been very few literature reports on the syntheses of Sb nanotubes by using without template-assisted or surfactant-assisted method. Therefore, it is highly desirable and significant, as well as a big challenge, to develop a facile route to directly synthesize the relatively straight and uniformly dispersed nanotubes of Sb.

Herein, we have synthesized relatively straight and uniform Sb nanotubes via a facile solvothermal method without any surfactants or templates. The synthesized Sb nanotubes are confirmed to be pure rhombohedral phase and show better crystallinity. The Sb nanotubes have middle-hollow, open-ended structures, and an average size of the diameter of about 50 nm and the length of about 350 nm. Based on the morphologies and structures, the formation mechanism of Sb nanotubes is discussed.

## Methods

### Synthesis of Antimony Nanotubes

All the chemical reagents used in this experiment are analytical grade. In our synthetic system, antimony chloride (SbCl_3_) as the Sb source was reduced to form Sb nanotubes via a solvothermal reduction by Zn powder at 200 °C for 10 h. Toluene was selected as the solvent because it is stable and can dissolve SbCl_3_. In a typical process, SbCl_3_ (521 mg) was dispersed in toluene (40 ml) under vigorous stirring at 5000 rad/min for 30 min. Subsequently, the mixed solution was transferred into a Teflon-lined stainless steel autoclave (50 mL), followed closely by the addition of zinc powers (75 mg). The autoclave was filled with the mixed solution up to 80 % of its total capacity, then sealed and maintained at 200 °C for 10 h. When the reaction was finished, the resultants were filtered off and rinsed with absolute alcohol, dilute hydrochloric acid, and deionized water for three times, respectively, then dried at 50 °C under vacuum.

### Characterization

The XRD patterns of the products were collected on a Bruker (AXS D8) X-ray diffractometer with Cu Kα radiation (λ = 1.5406 Å). An accelerating voltage of 40 kV and emission current of 30 mA were adopted for the measurements. The morphologies of as-synthesized samples were characterized by field emission scanning electron microscope (FE-SEM, Hitachi-4800, Japan) and high-resolution transmission electron microscope (TEM, FEI Tecnai G2 F20, 200 kV). The local chemical compositions of the samples were examined by energy-dispersive X-ray spectroscopy (EDX) performed in the transmission electron microscope.

## Results and Discussion

**Fig. 1:**
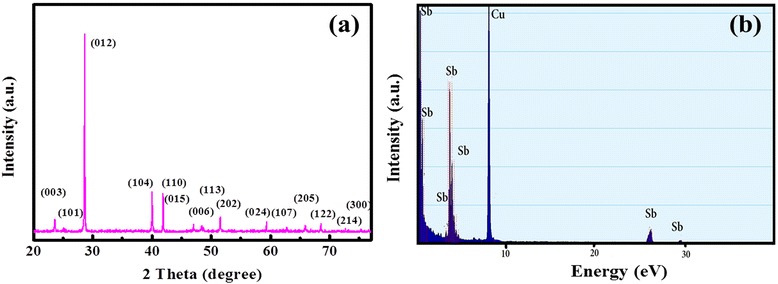
**a** The XRD pattern and **b** the EDX analysis of Sb nanotubes after filtered and rinsed

The XRD patterns of the powder samples are shown in Fig. 1a. All the diffraction peaks can be assigned to pure rhombohedral phase of Sb with lattice constants *a* = *b* = 4.307 Å, and *c* = 11.273 Å (JCPDS 85-1324), which is indicative of the formation of the Sb product from the complete reduction of Sb^3+^ by the Zn powder as reductant. And these peaks are sharp and well defined for the sample confirming the existence of pure phase crystalline Sb products. Furthermore, the EDX is performed to detect the chemical composition of as-obtained sample (Fig. 1b). From the spectrum, we can see that only Sb peaks are observed together with the Cu peak from the copper grid. And the mass ratio of Sb is 99.12 at.%, which also confirms the formation of the Sb and the purity of product.

**Fig. 2:**
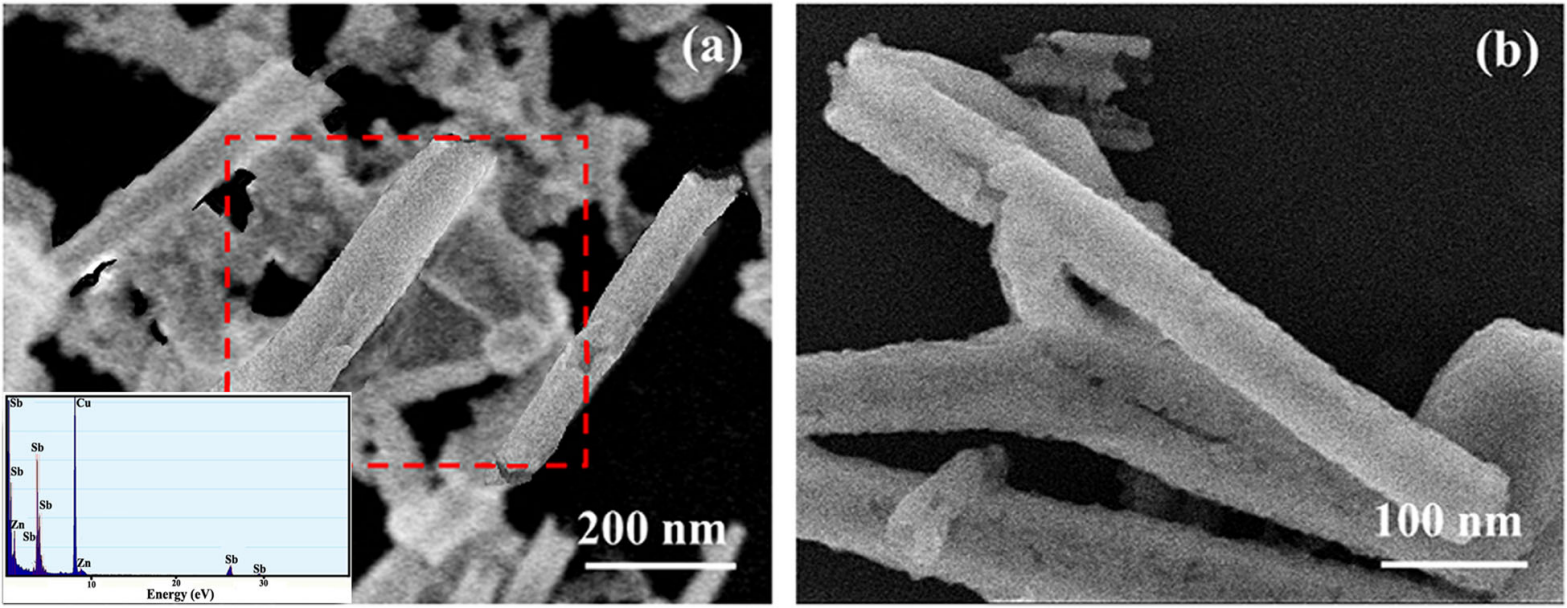
SEM images of **a** the original sample and **b** the sample with filtered and rinsed for Sb nanotubes, in which the inset in **a** shows the EDX pattern of the original sample

To observe the morphologies of as-synthesized samples, SEM images are shown in Fig. 2. The original sample without the unfiltered and unrinsed process shows not only the coexisting structures of tubular and lamellar Sb but also the clustered structures from unreacted Zn powers (Fig. 2a). The chemical composition of the sample also is detected by the EDX (Fig. 2a, insert), which indicates the original sample is composed of Sb (95.58 at.%) and Zn (2.44 at.%). Note that a half-rolling structure is displayed (the red dashed box in Fig. 2a), which suggests that the formation of Sb nanotube mainly depends on its lamellar structure. The similar results have been previously reported [6–9]. Compared with the original sample, the filtered and rinsed sample exhibits relatively neat and uniform tubular structures without the clustered phenomenon in Fig. 2b. The nanotubes have an average size of diameters of about 50 nm and lengths of about 350 nm. The above results illustrate that the filtered and rinsed post-processes are an important procedure for the purity of Sb nanotubes; thus, all of the samples have been precisely performing the post-processes before the characterization and analysis.

**Fig. 3:**
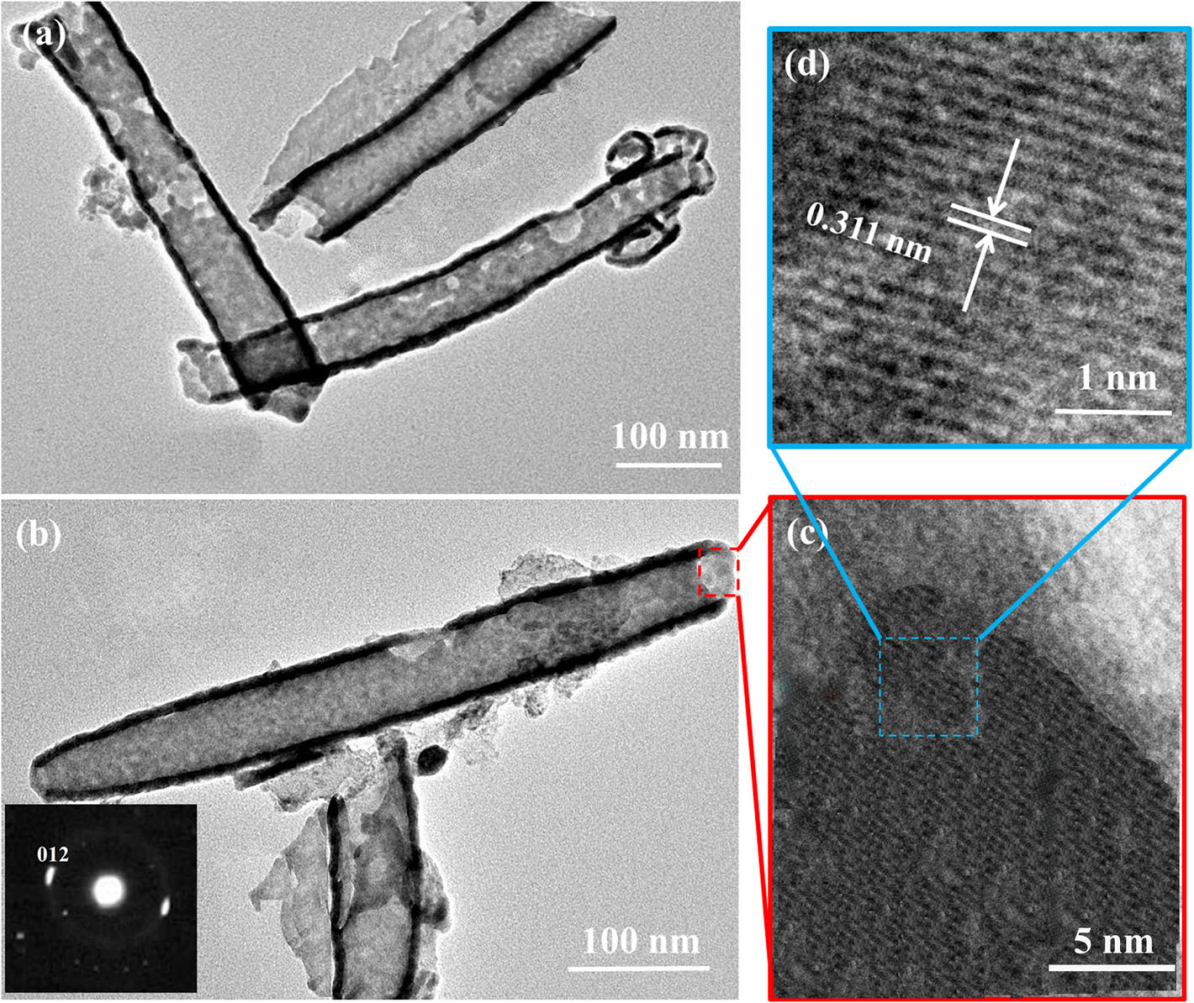
TEM images of **a** the coexisting with tubular and lamellar structures and **b** single nanotube for Sb. **c**, **d** HRTEM images of enlarge corresponding area of the top for single Sb nanotube, in which the inset in **b** shows the SAED pattern of the single Sb nanotube

TEM and HRTEM are used to further investigation of the detailed structure of the Sb nanotubes. The TEM image of Sb also demonstrate that the coexisting structures of tubular and lamellar of Sb (Fig. 3a), and the average size of Sb nanotubes is about 50 × 350 nm. To clearly observe tubular structure, the TEM image of an individual nanotube is selected (Fig. 3b), in which Sb nanotube has a middle-hollow, open-ended, and multi-walled structure. The wall thickness of Sb nanotube is about 10 nm. These morphologies indicate that Sb nanotube can be formed through the rolling up of regularly ordered molecular layers during the solvothermal treatment process, in which toluene as a solvent plays the role of a structure-directing agent and should provide a possible driving force to facilitate the rolling of lamellar structures at 200 °C for 10 h [20]. The selected area electron diffraction (SAED) pattern for the Sb nanotubes (Fig. 3b, insert) shows that brighter dots on the less bright ring can be indexed as the diffraction of (012) planes, indicative of the [012] radial direction of the Sb nanotubes. In addition, the HRTEM image of the enlarge corresponding area of the top of individual nanotube clearly exhibits that the interlayer spacing of the lattice planes for the Sb nanotube is about 0.311 nm (Fig. 3c, d), which is consistent with the previous reports [24].

**Fig. 4:**
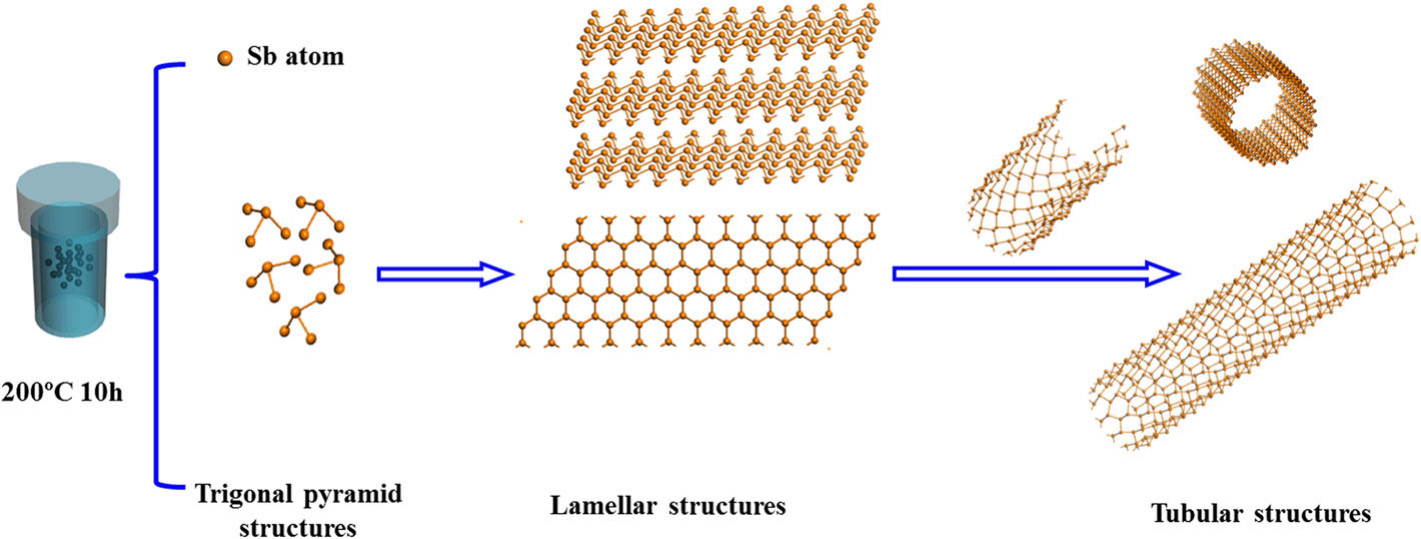
The schematic depiction of proposed formation mechanism for Sb nanotubes

Considering the coexisting of lamellar and tubular structures in our system, we propose that the formation of Sb nanotubes might also involve the rolling of its pseudolamellar structure, as shown in Fig. 4; the formation processes are as follows: First, some primary Sb atoms are formed by solvothermal reductive reaction at certain temperature and pressure. Second, the collision of these Sb atoms may provide the opportunity that each Sb atom is connected with three closest neighbors and thus forms trigonal pyramids by covalent bonds. These trigonal pyramids further form a folded Sb lamellar structure driven by the anisotropic growth tendency of Sb [10, 21]. In addition, the structures of these layers are puckered due to the presence of “lone pairs,” responsible for a weak van der Waals interaction between the adjacent layers [21]. When shaking in solvent, the Zn ions or solvent molecules may be intercalated into the adjacent layers to reduce van der Waals interaction at the edges of the layers and expand the spacing of neighboring layers during the heating process. As a result, the turbostratic restacked lamellar structures will become very easily cleaved into individual layer and roll into nanotube structures through driving force (Fig. 4c, d), similar to the formation of carbon nanotubes and other inorganic nanotubes for Bi, BN, and WS_2_ with the lamellar structures [5–9]. The driving force for the rolling of the layers can be generated due to one or more of the following reasons: (1) The asymmetric structures caused by hydrogen deficiency in surface layers, which may generate a asymmetric surface tension to drive the surface layer to cleave from the turbostratic restacked layers and to roll into tubular structures [25, 26]. (2) The thermal stress may initiate the rolling of the layers with reduced interlayer forces at the edges at high temperature [27]. (3) The mechanical tensions generated in the process of dissolution/crystallization of layers may be another driving force for the rolling of layers at high temperature and pressure. The difference of width between the layers can induce excess surface energy during the growth and crystallization of layers. In order to decrease the excess surface energy, the rolling structures of cleaved layers are formed [28, 29]. Since no template or other chemical surfactant was introduced in our study, the driving force may be in good agreement with (3) or (4) or the combined action of (3) and (4) at high temperature and pressure. As direct experimental evidences, the nanotube structures of half-rolled and wholly rolled lamellar layers can fully support the rolling formation of Sb nanotube from the above images of SEM and TEM. The formula of solvothermal reactions may be expressed as follows:

$$ 2{\mathrm{SbCl}}_3 + 3\mathrm{Z}\mathrm{n} = 2\mathrm{S}\mathrm{b} + 3{\mathrm{ZnCl}}_2 $$

However, our understanding of the formation mechanism for Sb nanotubes is limited during the solvothermal process. Therefore, further investigations would be necessary to clarify in detail the mechanism.

## Conclusions

Sb nanotubes were successfully synthesized via a facile solvothermal process without the need for any surfactants or templates. In the synthetic system, antimony chloride as the Sb source was reduced to form Sb nanotubes by using Zn powder in toluene solvent at 200 °C for 10 h. The XRD analysis confirms that the Sb nanotubes are pure rhombohedral phase. The images of SEM and TEM reveal that the samples are the coexisting structures of lamellar and tubular Sb, in which uniform Sb nanotubes have a middle-hollow, open-ended, and multi-walled structure. And the Sb nanotubes have an average size of about 50 × 350 nm and the wall thickness of about 10 nm. On the basis of the structural and morphological studies, a possible rolling formation mechanism is proposed to explain the formation of Sb nanotubes. It is expected that uniform Sb nanotubes can further be used in wide applications.
